# Development and characterization of an injectable thermoresponsive PLGA nanoparticle-loaded *in situ* gel for sustained exemestane delivery

**DOI:** 10.3389/fonc.2026.1905253

**Published:** 2026-07-23

**Authors:** Priya Singh, Namrata Swain, Shagufa Shamim, Vemuri Sai Phanindra, Sruti Sikha Dikhit

**Affiliations:** School of Pharmacy, GITAM (Deemed to be) University, Hyderabad, Telangana, India

**Keywords:** breast cancer, exemestane, HET-CAM assay, *in situ* gel, PLGA nanoparticles

## Abstract

**Introduction:**

The present study aimed to develop and evaluate a thermoresponsive in situ gel incorporating Exemestane-loaded poly(lactic-co-glycolic acid) (PLGA) nanoparticles for sustained drug delivery.

**Methods:**

Exemestane-loaded PLGA nanoparticles were prepared by nanoprecipitation and characterised for particle size, zeta potential, morphology, entrapment efficiency, and drug release behaviour.

**Results:**

The nanoparticle exhibited a mean particle size of 257.6 nm, moderate stability with a zeta potential of–15.3mV, high encapsulation efficiency (93.46± 2.46%) and drug loading 27.8±1.2% (0.278±0.012mg Exemestane /mg nanoparticles), indicating successful formulation. The optimised nanoparticles were incorporated into a poloxamer-based thermoresponsive in situ gel system modified with chitosan to enhance gel strength and bioadhesion. The formulation was optimised using a Box–Behnken design, achieving a gelation temperature of 38.3±0.5 °C and gelation time of 0.75±0.5 min, suitable for physiological conditions. In vitro drug release studies demonstrated a sustained release profile with minimal burst effect, achieving approximately 80% drug release over 24 hours. Release kinetics followed the Korsmeyer–Peppas model, indicating an Fickian and diffusion mechanism governed by both diffusion and polymer erosion.The formulation exhibited desirable physicochemical properties, appropriate rheological behaviour, controlled gel erosion, and stability over three months.Ex vivo and HET-CAM studies indicated reduced angiogenesis, suggesting potential therapeutic efficacy in breast cancer treatment.

**Discussion:**

Overall, the developed nanoparticle-loaded thermoresponsive in situ gel represents a promising localised sustained drug delivery platform for Exemestane and warrants further evaluation in appropriate cellular and in vivo breast cancer models.

## Graphical Abstract

**Figure:**
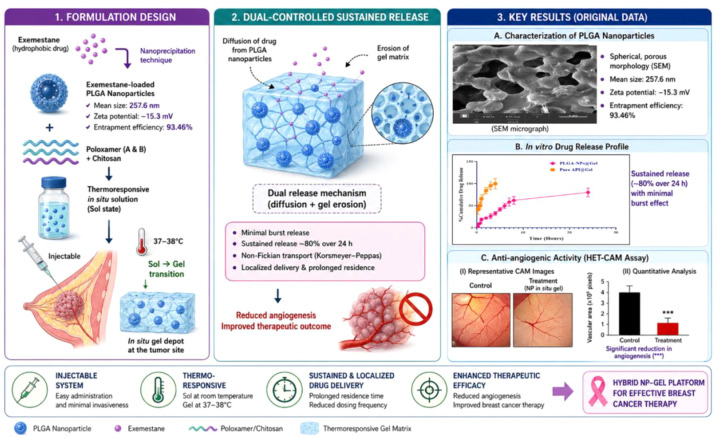

## 1 Introduction

Drug delivery systems play an important role in the therapeutic performance of poorly soluble drugs and chronically administered drugs. Traditional dosage forms often fail to maintain the therapeutic effect for a longer period of time at the target site itself, leading to multiple dosing, systemic toxicity and reduced patient compliance. Then, to reduce these complications, controlled and sustained delivery platforms are essential to increase drug residence time in the body, minimise dose and dosing frequency, and minimise adverse effects, particularly in long-term anticancer treatment (1–3).

Polymeric nanoparticles have been extensively investigated as versatile carriers for sustained drug delivery. Among them, poly(lactic-co-glycolic acid) (PLGA) nanoparticles are widely preferred due to their biodegradability, biocompatibility, and regulatory acceptance (4). PLGA nanoparticles offer controlled drug encapsulation and release, protection of labile molecules, and tunable release kinetics. However, nanoparticle formulations administered as suspensions may exhibit limitations, including initial burst release, rapid diffusion away from the site of administration, and reduced local retention, which can compromise sustained delivery performance (5). To overcome these problems of drug delivery, injectable *in-situ* gelling systems have received more attention as localised drug delivery systems. The temperature-responsive *in situ* gels undergo a sol-gel transition upon exposure to physiological temperatures, promoting minimal invasiveness during administration and prolonged residence time at the injection site (6). Poloxamer-based temperature-sensitive gelling systems are often modified with bioadhesive polymers, such as chitosan, to improve injectability, gel strength, and biocompatibility, as well as to achieve sustained release, as reported in this research (5, 6). The combination of PLGA nanoparticles with thermoresponsive *in-situ* gels represents a rational and advanced delivery strategy that synergistically integrates the advantages of both systems. Incorporation of nanoparticles into a gel matrix creates a hybrid platform capable of offering dual-controlled release, governed by nanoparticle diffusion and gel erosion mechanisms (7, 8). This approach not only reduces burst release but also enhances drug localisation and prolongs release duration, thereby improving delivery efficiency. Injectable thermoresponsive *in situ* gels offer a promising strategy for localized breast cancer treatment due to their ability to undergo sol-to-gel transition at physiological temperature following administration. The formulation can be injected as a free-flowing solution and rapidly forms a gel depot at the target site, enabling prolonged local drug retention and sustained release while minimizing systemic drug exposure. Incorporation of drug-loaded PLGA nanoparticles within the gel further provides dual-controlled release, thereby enhancing therapeutic efficacy, reducing dosing frequency, and potentially improving the management of residual tumor cells following surgical resection (9).

Such nanoparticle-loaded *in-situ* gel systems are increasingly being explored for the controlled delivery of hydrophobic drugs that require sustained release profiles. Exemestane, a hydrophobic aromatase inhibitor, presents formulation challenges related to solubility and sustained delivery (10). Encapsulation of Exemestane into PLGA nanoparticles, followed by their incorporation into a thermoresponsive *in-situ* gel, offers a promising delivery-oriented solution to overcome these limitations (11). In the present study, a thermoresponsive *in situ* gel loaded with Exemestane-encapsulated PLGA nanoparticles was developed and evaluated as a sustained drug-delivery system. The formulation was characterised for nanoparticle properties, gelation behaviour, injectability, and release performance, along with *in vitro* and *ex vivo* drug release studies to assess its sustained-release potential.

The novelty of this work lies in the rational design of a hybrid drug delivery system comprising Exemestane-loaded PLGA nanoparticles embedded in a thermoresponsive *in-situ* gel matrix to achieve prolonged, localised drug release. Unlike standalone nanoparticle or gel formulations, the developed system provides a dual-controlled delivery mechanism, combining nanoparticle-mediated release with gel-based diffusion control to minimise burst release and enhance retention. To the best of our knowledge, limited reports have focused on the delivery-system-driven integration of PLGA nanoparticles into thermosensitive *in-situ* gels for sustained Exemestane release supported by both *in vitro* and *ex vivo* evaluation. This study highlights the potential of nanoparticle-loaded *in-situ* gels as an advanced platform for sustained drug delivery applications.

## 2 Materials and methods

### 2.1 Materials

PLGA (Resomer RG 653 H) was a gift sample procured from Evonik Industries Pvt Ltd. Poloxamer 407 and poloxamer 188 were purchased from Hi Media Pvt Ltd. Exemestane was purchased from Sigma Pvt. Ltd (Mumbai). All the remaining substances were of analytical grade.

### 2.2 Methodology

#### 2.2.1 Preparation of nanoparticles

Exemestane-loaded poly(lactic-co-glycolic acid) (PLGA) nanoparticles were prepared using the nanoprecipitation (solvent displacement) method with slight modifications. The composition of the formulations used for nanoparticle preparation is presented in **Table 1**. Briefly, the organic phase was prepared by dissolving 50 mg of PLGA (5 mg/mL) and 20 mg of Exemestane (2 mg/mL) in 10 mL of acetone, maintaining a drug-to-polymer ratio of 20:50 (w/w). This ratio was selected based on previous literature reports to achieve high drug encapsulation while maintaining desirable nanoparticle characteristics.The aqueous phase consisted of 10 mL of distilled water containing 0.8% (w/v) polyvinyl alcohol (PVA) as a stabilizer. The PVA solution was stirred until completely dissolved and maintained at room temperature before nanoparticle preparation. The organic phase was added dropwise into the aqueous phase at a controlled rate of approximately 0.5 mL/min using a micropipette under continuous magnetic stirring at 700 rpm. Following complete addition, the resulting nanosuspension was stirred continuously to facilitate complete evaporation of acetone, leading to the spontaneous formation of Exemestane-loaded PLGA nanoparticles. The nanoparticle suspension was subsequently centrifuged at 15,000 rpm (≈21,000 × g) for 20 min at 4 °C. The collected nanoparticles were washed three times with Milli-Q water to remove unentrapped drug and residual PVA, followed by redispersion in distilled water for further characterization. The purified nanoparticles were frozen at −80 °C overnight and lyophilized using a laboratory freeze dryer at a condenser temperature of −50 °C under a vacuum of 0.05 mbar for 48 h. The freeze-dried nanoparticles were stored at 4 °C until further characterization and formulation studies. Blank PLGA nanoparticles were prepared following the same procedure without the addition of Exemestane and were used as control formulations (12).

**Table 1:** Concentration of raw materials used for the development of Nanoparticles.

Formulation	Concentrations		
	PLGA (mg/ml)	PVA (%w/v)	EXE
PLGA-NPs	5	0.8	–
EXE-PLGA-NPs	5	0.8	2 mg/mL

#### 2.2.2 Characterisation of PLGA nanoparticles

##### 2.2.2.1 Particle size, zeta potential & morphological analysis

Dynamic light scattering (DLS) was used to quantify particle size and ζ-potential using a Zetasizer (Horiba SZ-100 Z2) at 25 °C. Nanoparticle suspensions were diluted 1:100 (v/v) with Milli-Q water before DLS measurements. The morphological properties of PLGA nanoparticles suspended in the original medium were investigated using a Field Emission Scanning Electron Microscope (FESEM) (MIRA S6123). A drop of each sample was deposited on copper grids (300 mesh) and dried before being visualised (12).

##### 2.2.2.2 Per cent yield, entrapment efficiency & drug loading

Following nanoparticle preparation, the suspension was centrifuged at 15,000 rpm for 20 min at 4 °C to separate the nanoparticles from the aqueous medium. The collected nanoparticle pellet was washed three times with Milli-Q water to remove residual surfactant and unencapsulated drug. After the final washing step, the nanoparticles were frozen at −80 °C overnight and subsequently lyophilized at −50 °C under a vacuum of 0.05 mbar for 48 h until a constant weight was obtained. The dried nanoparticles were accurately weighed using an analytical balance (accuracy ±0.1 mg). The percentage yield was calculated as the ratio of the weight of the recovered dried nanoparticles to the total initial weight of the polymer and drug used for formulation according to the following equation:


$$\%\;Y=\frac{Produced\;NPs\;amount\left(mg\right)}{Drug\left(mg\right)+amount\;of\;PLGA\left(mg\right)\;}\times 100$$

The collected supernatant was appropriately diluted with phosphate buffer (pH 7.4), and the concentration of free Exemestane was determined spectrophotometrically at 253 nm using a previously established calibration curve (2–20 μg/mL). The encapsulation efficiency was calculated using the following equation:


$$\%EE=\frac{Total\;drug\;amount\left(mg\right)-Free\;drug\;amount\left(mg\right)}{Total\;drug\;amount\left(mg\right)}\times 100$$

The amount of encapsulated drug was calculated by subtracting the amount of free drug present in the supernatant from the total amount of drug initially added during nanoparticle preparation (13).

Drug loading of the optimized Exemestane-loaded PLGA nanoparticles was determined after completion of the encapsulation efficiency study (14). Following centrifugation, washing, and lyophilization, the recovered nanoparticles were accurately weighed. Drug loading (DL) was calculated according to the following equation:


$$\text{DL}\left(\%\right)=\frac{\text{Amount of Exemestane}\left(\text{mg}\right)}{\text{Weight of recovered nanoparticles}\left(\text{mg}\right)}\times 100$$

All measurements were performed in triplicate (n = 3), and the results are presented as mean ± standard deviation.

##### 2.2.2.3 Fourier transform infrared spectroscopy

Fourier Transform Infrared (FTIR) spectroscopy was performed using a Bruker ALPHA II FTIR spectrometer (Bruker Optics, Germany) equipped with an attenuated total reflectance (ATR) accessory. Spectra of pure Exemestane, PLGA, Poloxamer 127, and the optimized Exemestane-loaded PLGA nanoparticles were recorded in ATR mode over the spectral range of 4000–400 cm^−1^ with a spectral resolution of 4 cm^−1^. Each spectrum was obtained by averaging 32 consecutive scans at room temperature. Background spectra were recorded before each measurement and automatically subtracted. The obtained spectra were analyzed to identify characteristic functional groups and to evaluate any possible drug–polymer interactions or chemical incompatibilities following nanoparticle formulation (15, 16).

##### 2.2.2.4 P-XRD analysis

XRD is used to determine whether a drug is crystalline or amorphous in the solid state. This nature will define the solubility and bioavailability of the compounds. X-ray diffraction of the pure drug, excipients, and optimised NPs was performed using a powder X-ray diffractometer (MINIFLEX 600). The glass slide containing the dry powdered materials under evaluation was put on the X-ray diffractometer. Within a 2θ range of 10 to 90°, the scanning rate was 10 minutes (17).

##### 2.2.2.5 TGA-DTA analysis

TGA and DTA were performed to evaluate the thermal stability of PLGA nanoparticles. Lyophilised samples (5–10 mg) were placed in an alumina crucible and analysed using a simultaneous thermal analyser (STA 2500) under a nitrogen atmosphere. The samples were heated from room temperature to 600 °C at a heating rate of 10 °C/min. Weight loss and thermal transitions were recorded as a function of temperature (18).

##### 2.2.2.6 *In vitro* drug release

The dialysis bag diffusion method was used to investigate the *in vitro* drug release of PLGA nanoparticle compositions. A dialysis bag containing 5 ml of drug-loaded nanoparticles was put in a beaker with 100 ml of pH 7.4 phosphate buffer. Dialysis membrane (MWCO 12–14 kDa) was pre-soaked overnight before use. Throughout the experiment, the beaker was kept at 37 ± 1 °C on a magnetic stirrer. The rpm was kept at 100 rpm throughout the experiment. At predetermined intervals, samples (2 ml) were removed and replaced with equivalent volumes of brand-new pH 7.4 phosphate buffer (19, 20). After suitable dilutions, the samples were analysed using a UV–Visible spectrophotometer at 253 nm (21).

#### 2.2.3 Formulation and optimisation of *in-situ* gel

A thermoresponsive *in-situ* gel was formulated using Poloxamer 407 (Poloxamer A) and Poloxamer 188 (Poloxamer B) in combination with chitosan. Poloxamer 407 and Poloxamer 188 (at the concentrations listed in **Table 2**) were dissolved in 10 mL of purified water using the cold method and then hydrated overnight at 4 °C until a clear solution was obtained. Chitosan (2% w/v) was separately dissolved in 2% v/v acetic acid under continuous stirring to obtain a uniform solution. The previously prepared PLGA nanoparticle suspension was then mixed with the chitosan solution under gentle stirring to ensure homogeneous dispersion of nanoparticles. The nanoparticle–chitosan mixture was gradually added to the cold poloxamer solution under mild stirring to obtain a uniform nanoparticle-loaded *in-situ* gel formulation. The final formulation was stored at 4 °C until further evaluation (22).

**Table 2:** Experimental design and optimisation of thermoresponsive in-situ gel formulations illustrating the influence of Poloxamer A (%), Poloxamer B (%), and stirring time on gelation time and gelation temperature.

Runs	Poloxamer A (%)	Poloxamer B (%)	Stirring time (min)	Gelation time (min)	Gelation temp. (°C)
1	24.6818	4	17.5	2.5	40
2	23	4	17.5	2.02	45
3	23	4	21.7045	1.17	49
4	21.3182	4	17.5	3.5	55
5	24	3	20	0.92	40
6	24	5	20	1.55	39.5
7	23	4	17.5	2.02	40
8	22	3	15	3.5	56
9	23	5.68179	17.5	2.7	41
10	23	4	13.2955	3.5	36
11	22	3	20	1.88	38
12	22	5	20	3.03	47.5
13	23	4	17.5	2.02	40
14	23	2.31821	17.5	1.67	36.9
15	24	3	15	1.17	36
16	23	4	17.5	0.27	35
17	22	5	15	2.15	40
18	23	4	17.5	2.02	39.5
19	23	4	17.5	2.02	43
20	24	5	15	1.87	36.9

A Box-Behnken design was used to optimize PLGA-NPs loaded *in situ* gel, and a three-factor, three-level design was used to examine the correlations between independent factors and dependent variables (**Table 2**). The concentration of Poloxamer A (%) (X1), Poloxamer B (%) (X2), and Stirring Time (%) (X3) were the chosen independent variables taken into consideration. On the other hand, these variables’ three levels were coded as −1, 0, and +1 for low, medium, and high, respectively. The dependent variables chosen were Gelation Temperature (Y2) and Gelation Time (Y1). To examine how each level interacted with formulation variables, twenty formulations were needed. The statistical validity of the produced polynomial equations was evaluated using ANOVA. All observed responses were simultaneously fitted to various models. The best-fitting experimental model (main, interaction, and quadratic) was chosen after comparing numerous statistical metrics, such as the CV (coefficient of variation), R2 (multiple correlation coefficient), adjusted R2 (Adjusted R2), and anticipated R2. The level of significance was chosen at p-value< 0.05 (23).

#### 2.2.4 Characterisation of *in-situ* gel

##### 2.2.4.1 Visual appearance, pH, gelling capacity and drug content

The formulation was evaluated on its overall appearance (color and clarity). The pH of the created *in situ* gels was determined using a previously calibrated portable digital pH meter (Hanna Instruments, USA). To evaluate the drug content, 1 ml of the *in situ* gel was diluted with 100 ml of methanol for 5 minutes using a laboratory vortex mixer (Witeg, Germany). To remove particulate matter, the solution was filtered through an AXIVA^®^ All Pure Syringe Filter (China) with a pore size of 0.45 µm. The exemestane content in the filtered solution was then quantified using a UV-visible spectrophotometer (Shimadzu 1280, Japan) at 253 nm (24). The gelling capacity of the nanoparticle-loaded *in-situ* gel formulation was evaluated using a calcium chloride–induced gelation method. Briefly, a calcium chloride solution was prepared by dissolving 0.55 g of CaCl_2_ in 50 mL of distilled water at room temperature. The *in-situ* gel formulation was then added dropwise into the calcium chloride solution using a syringe under static conditions. The formation of gel droplets was visually observed and recorded. The gelling capacity was assessed by measuring the time to gel formation and the stability of the formed gel droplets in the medium. The formulation was graded as “++” when immediate gel formation was observed, and the gel droplets remained intact and stable for an extended period without disintegration. All measurements were performed in triplicate to ensure reproducibility.

##### 2.2.4.2 Viscosity

Viscosity measurements were conducted with a DV Next Brookfield viscometer equipped with a thermostatic circulator to maintain a consistent temperature during testing. A cone-plate geometry with a radius of 49.9 mm, a gap of 0.052 mm, and an angle of 1° was used, with a shear rate range of 0.01 to 100 s. Measurements were taken in triplicate at 38 °C and reported as means ± SD (25).

##### 2.2.4.3 Gelation temperature

To evaluate the gelation temperature of a thermosensitive *in situ* gel, 2 mL aliquots were transferred to a glass test tube and placed in a temperature-controlled water bath. The temperature of the water bath was steadily increased by 2 °C every 5 minutes, beginning at 25 °C, until the gel reached a phase transition. This phase transition is characterised by a significant increase in viscosity, which corresponds to the gelation temperature, also known as the critical solution temperature. The sample was next assessed for gelation, which was determined when the meniscus remained stationary and did not shift after tilting the test tube to 90 degrees (26).

##### 2.2.4.4 *In vitro* gelation time

The gelation time of several *in situ* gel formulations using poloxamer as the gelling agent was measured using the test tube inversion method, as previously described. Two millilitres of the produced solutions were incubated at 37 °C in a temperature-controlled water bath. The test tubes were changed over every 30 seconds to determine the flowability of the liquids. The gelation time was defined as the time required to generate a gel that did not flow within 5 seconds of inverting the test tube (27).

##### 2.2.4.5 Rheological studies

The MCR 301e rheometer was used to do dynamic rheological measurements. The tests were carried out in a parallel-plate configuration (top plate diameter: 20 mm, gap: 1 mm). The viscosity curve was obtained in CR (Controlled Rate) mode at 25 °C. Storage and loss moduli were evaluated in CS (controlled stress) mode at 37 °C with a constant frequency of 1.000 Hz (28, 29).

##### 2.2.4.6 FTIR analysis (compatibility)

The FT-IR spectra of NPs were obtained using an infrared spectrometer (Bruker ALPHA II 211515) spanning the wavelength range of 4000–500 cm-1. Pure excipients, pure drugs, and the optimized formulation were also examined as references (17).

##### 2.2.4.7 P-XRD

The same procedure has been followed as described for the PLGA-NPs characterisation section 2.2.2 (17).

##### 2.2.4.8 TGA-DTA analysis

The thermal behaviour of the *in-situ* gel formulations was investigated using thermogravimetry and differential thermal analysis (TGA-DTA) (STA 2500 “Regulus” model). Approximately 3 mg of each sample was placed in an aluminum pan, sealed, and heated from 0 to 600 °C at a rate of 10 °C per minute. TGA-DTA thermograms were compared to discover significant changes caused by the interaction of drugs with polymers in formulations.

##### 2.2.4.9 FESEM analysis

FESEM is a high-resolution imaging technique used to investigate the surface morphology of materials. FESEM (B2062-152-MIRA) was used to examine freeze-dried samples of *in-situ* gel compositions in comparison to their separate pure components. The samples were mounted on an aluminum stub with double-sided carbon tape, and the sample was placed on the tape using a sterile spatula. The tip setup was sputter-coated with a thin gold film to ensure conductivity for displaying three-dimensional images of the samples. Imaging was performed at 10 kV, with images recorded at magnifications ranging from 200 to 20,000X (25).

##### 2.2.4.10 *In vitro* drug release and release kinetic study

The *in vitro* drug release tests were carried out using a dialysis membrane (cut-off: 12–14 kDa) immersed in phosphate buffer (pH 7.4). A 50-mL beaker was filled with 30 milliliters of phosphate buffer (pH 7.4). A dialysis bag was filled with precisely 1 mL of the *in-situ* gel formulation (containing 5mg/mL of Exe) and sealed at both ends. The sample bag was immersed in the buffer. The complete setup was placed on a thermostatic magnetic stirrer, swirled constantly at 100 rpm, and kept at 37 °C for 24 hours. To maintain stable sink conditions, the drug-releasing samples were collected every hour and replenished with 2 mL of fresh buffer. The collected samples were measured at 253 nm for exemestane using a UV–visible spectrophotometer (Shimadzu UV 1800, USA). All sample experiments were conducted in triplicate and compared with the *in vitro* release profile of unprocessed drug samples containing an equivalent drug concentration (30, 31). Data obtained from the *in vitro* dissolution study were further investigated for release kinetics using the DDSolver software program (32).

##### 2.2.4.11 Texture analysis

Texture analysis of the optimised thermoresponsive *in situ* gel was performed using a Texture Analyzer (TA10/490L) to evaluate the mechanical properties of the formulation, including hardness, adhesiveness, and cohesiveness. Briefly, an appropriate quantity of the gel formulation was transferred into a cylindrical sample holder and equilibrated at 38 ± 0.5 °C to allow complete gelation under physiological conditions before analysis. A cylindrical probe was lowered vertically into the gel sample at a predetermined speed until a predetermined depth was reached, then withdrawn at the same speed, generating a force–time profile. The test was performed using a two-cycle compression mode (texture profile analysis) to simulate repeated deformation of the gel matrix.

##### 2.2.4.12 Gel erosion study

The gravimetric approach was used to analyze gel erosion, which involves the gel matrix degrading or disintegrating under physiological conditions, releasing the monomer or medication. It is critical to determine the mechanical strength of the *in situ* gel compositions. The formulations were stable at normal pH and body temperature. A 5 gram sample was placed in a 20 mL amber glass vial, weighed, and stored at 37 °C. The material was then treated with a 1 mL volume of 0.133 M Sorensen’s phosphate buffer (pH 7.2). At 30-minute intervals, the exposed buffer was removed and replaced with fresh buffer in the gel sample. After removing the buffer, the remaining gel was weighed to determine mass loss from erosion (30).

##### 2.2.4.13 Hen’s egg test-chorioallantoic membrane assay

An automatic rotating incubator (local market) with controlled temperature (37 °C) and relative humidity (45-65%) was utilized for incubating fertilised, freshly collected White Leghorn eggs measuring between 50 and 60 g (33). The test compounds were then applied to the CAM surface using 200 microliters at ambient temperature. The evaluation contained two experimental groups: 0.1 M NaOH solution (positive control) and the treatment group (*In situ* gel loaded with PLGA-NPs). The membrane and blood vessels were examined at specific time points (0, 0.5, 2, 5 minutes), and scores were recorded for irritation-induced haemorrhage (vessel bleeding), lysis (vessel integration), and coagulation (extra- and intravascular protein denaturation). The *in situ* gel contained PLGA nanoparticles equivalent to 2 mg/mL Exemestane. The final Exemestane concentration in the thermoresponsive *in situ* gel was fixed at 2 mg/mL based on previously reported thermoresponsive nanocarrier formulations for hydrophobic anticancer drugs and formulation considerations aimed at achieving homogeneous drug distribution, suitable injectability, physiological gelation behavior, and sustained drug release. This concentration produced a stable formulation without visible drug precipitation and was therefore selected for further characterization (34). The CAM images were captured using a Trinocular Inverted Microscope TS2FL, coupled with the Digital Camera Model DSFl3 and analysed using ImageJ software (35).

##### 2.2.4.14 Stability studies

Stability studies were performed on the *in situ* gel formulation in accordance with International Conference on Harmonisation requirements. Bottles of *in situ* gel were stored in a desiccator (Sabar Scientific, India) with a saturated sodium chloride solution (75 ± 5% RH). The desiccator was placed in a hot air oven maintained at 40 ± 2 °C, and samples were extracted after 1, 2, and 3 months. The appearance, drug content, gelation temperature, and gelation time of stored formulations were examined. The average values from the three determinations were recorded (36).

##### 2.2.4.15 Statistical analysis

All experiments were conducted in duplicate. The findings were analyzed using GraphPad Prism 9.0 software and Origin 2021, and presented as mean ± standard deviation (37, 38).

## 3 Results

### 3.1 Characterization of PLGA nanoparticles

#### 3.1.1 Particle size, zeta-potential and morphological analysis

**Figure 1:**
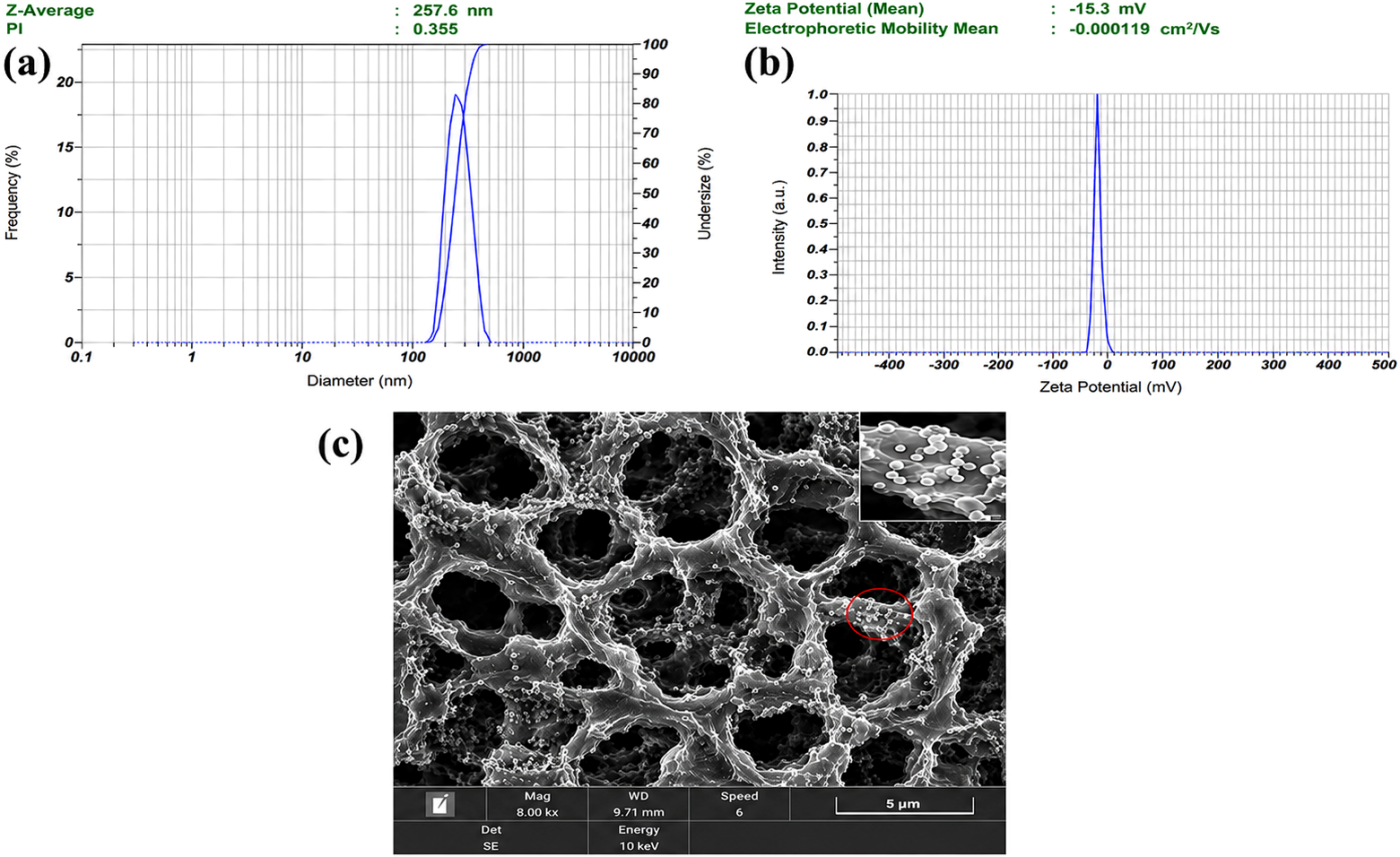
Physicochemical characterization of Exemestane-loaded PLGA nanoparticles showing **(a)** particle size distribution, **(b)** zeta potential profile, and **(c)** FESEM micrograph of freeze-dried Exemestane-loaded PLGA nanoparticles showing aggregated nanoparticle clusters formed during lyophilization. The inset highlights the spherical morphology of individual nanoparticles. The larger voids correspond to interparticle spaces between nanoparticle aggregates rather than pores within individual nanoparticles.

The prepared Exemestane-loaded PLGA nanoparticles were characterized for particle size, surface charge, and morphological properties to confirm successful nanoparticle formation and stability (**Figures 1a–c**). Particle size analysis using dynamic light scattering (**Figure 1a**) showed a Z-average particle size of 257.6 nm with a polydispersity index (PDI) of 0.355, indicating formation of nanoparticles within the desired nanometric range and possessing acceptable size distribution. Nanoparticles below 300 nm are advantageous for drug delivery applications as they provide increased surface area, improved drug encapsulation efficiency, and controlled drug release characteristics. The moderate PDI value suggests relatively uniform particle population, confirming effective emulsification and stabilization during nanoparticle preparation (39). Zeta potential measurement (**Figure 1b**) revealed a mean surface charge of –15.3 mV, indicating moderately stable colloidal dispersion. The negative surface charge is attributed to terminal carboxyl groups present in the PLGA polymer backbone. Although higher absolute values (> ± 30 mV) generally indicate strong electrostatic stabilization, PLGA nanoparticles stabilized using surfactants or poloxamers mainly rely on steric stabilization, which prevents particle aggregation despite moderate zeta potential values. Therefore, the obtained surface charge confirms sufficient nanoparticle stability suitable for further formulation development. Morphological evaluation by Scanning Electron Microscopy further confirmed nanoparticle characteristics. Morphological evaluation by field-emission scanning electron microscopy (FESEM) confirmed the successful formation of Exemestane-loaded PLGA nanoparticles. Field-emission scanning electron microscopy (FESEM) was performed to evaluate the surface morphology of the optimized Exemestane-loaded PLGA nanoparticles (**Figure 1C**). The FESEM micrograph revealed that the nanoparticles formed an interconnected porous aggregate after lyophilization, which is a common phenomenon resulting from particle aggregation during solvent removal and freeze-drying. The observed micron-sized voids represent interparticle spaces within the aggregated nanoparticle network rather than pores present within individual nanoparticles. Higher-magnification examination (inset) revealed that the aggregates were composed of discrete, nearly spherical nanoparticles with slight surface irregularities. These observations are consistent with the DLS results, which showed a mean hydrodynamic diameter of 257.6 nm. The apparent difference between FESEM and DLS measurements is expected because DLS measures hydrated nanoparticles dispersed in suspension, whereas FESEM visualizes dehydrated nanoparticle aggregates following sample preparation under high vacuum conditions (40, 41).

#### 3.1.2 Percent yield & entrapment efficiency

The percentage yield of the prepared PLGA nanoparticles was found to be high (83.96 ± 6.89%), indicating efficient recovery and minimal loss during the formulation process. This can be attributed to optimized processing conditions, including polymer concentration and solvent evaporation technique. The optimized formulation exhibited an encapsulation efficiency of 93.46 ± 2.46% with a corresponding drug loading of 27.8 ± 1.2% (0.278 ± 0.012 mg Exemestane/mg nanoparticles), indicating efficient incorporation of Exemestane within the PLGA polymeric matrix. The high entrapment efficiency is likely due to the hydrophobic nature of both the drug and PLGA, which enhances drug–polymer affinity and reduces drug leakage into the external phase during nanoparticle formation (42).

#### 3.1.3 FTIR analysis

**Figure 2:**
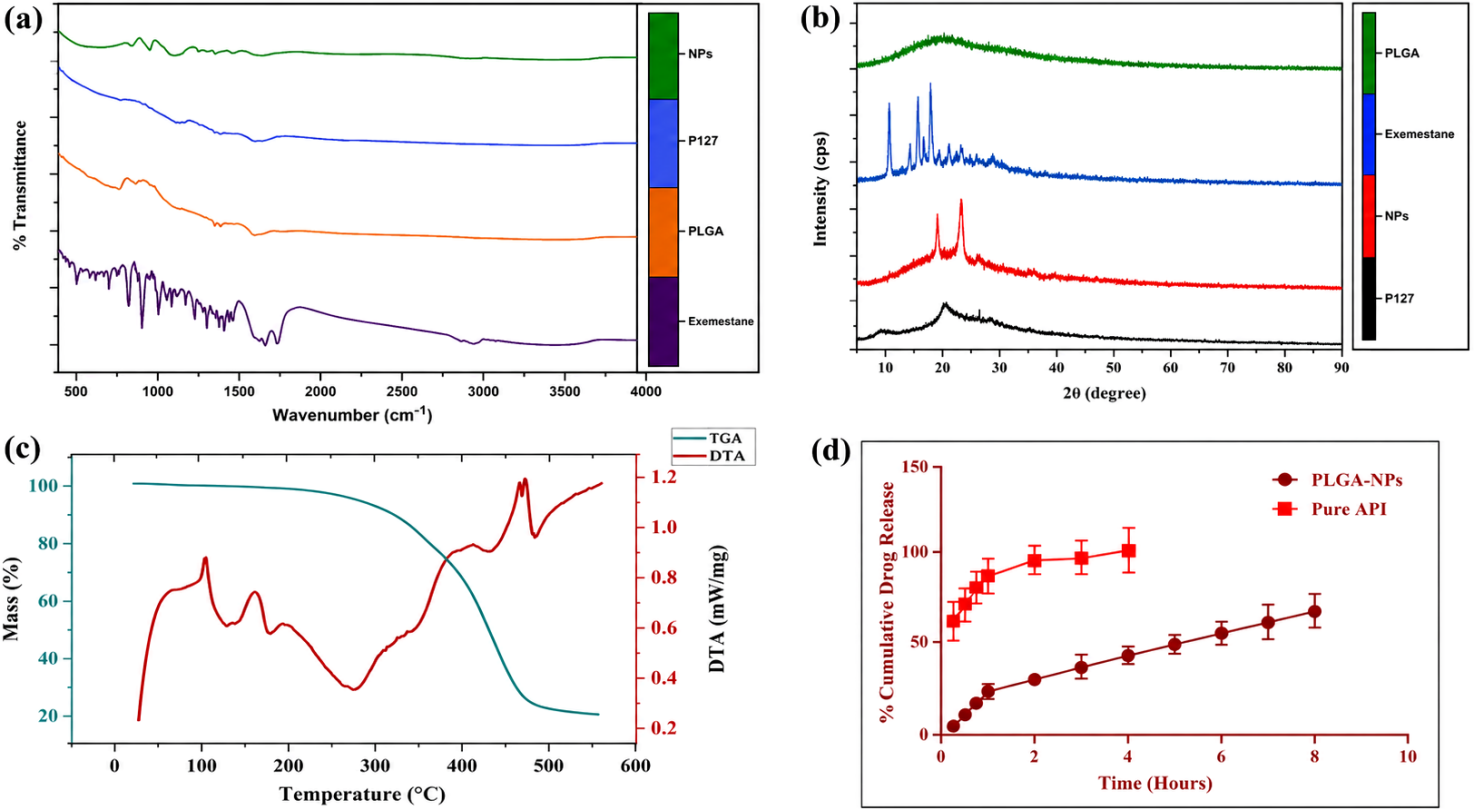
Characterization of Exemestane-loaded PLGA nanoparticles showing **(a)** FTIR spectra confirming drug–polymer compatibility, **(b)** P-XRD patterns demonstrating reduced crystallinity of the encapsulated drug, **(c)** TGA-DTA thermograms indicating thermal stability of the nanoparticle formulation, and **(d)** comparative in vitro drug release profile of pure Exemestane and Exemestane-loaded PLGA nanoparticles showing sustained release behavior.

Fourier Transform Infrared (FTIR) spectroscopy was performed to evaluate the compatibility between Exemestane and formulation excipients (PLGA and Poloxamer 127) and to confirm the absence of chemical interactions during nanoparticle preparation (**Figure 2a**). The FTIR spectrum of pure Exemestane exhibited characteristic absorption peaks corresponding to its functional groups (17). A prominent peak observed around ~1730 cm^−1^ corresponds to C=O stretching vibration of the ketone group, confirming the steroidal carbonyl functionality of Exemestane. Peaks appearing in the region of 2950–2850 cm^−1^ represent aliphatic C–H stretching vibrations, while bands observed near 1450–1600 cm^−1^ are attributed to C=C stretching of the steroidal backbone. Additional peaks between 1100–1250 cm^−1^ correspond to C–O stretching vibrations (43). The FTIR spectrum of PLGA showed characteristic ester carbonyl stretching around 1750 cm^−1^, along with C–O–C stretching vibrations in the region of 1080–1180 cm^−1^, confirming the polymeric ester structure. Similarly, The characteristic absorption bands of Poloxamer corresponding to O–H stretching (~3400 cm^−1^) and aliphatic C–H stretching (~2880 cm^−1^) are generally reported in the literature. In the present spectrum, these bands were not distinctly resolved because of their broad nature and overlap with neighboring absorption bands. However, the remaining characteristic peaks were consistent with the reported FTIR profile of Poloxamer, confirming its presence in the formulation (44). The FTIR spectrum of the Exemestane-loaded PLGA nanoparticles (NPs) retained all major characteristic peaks of the drug and excipients without the appearance of new peaks, peak disappearance, or significant peak shifting. The characteristic carbonyl peak of Exemestane remained identifiable within the nanoparticle spectrum, although with slight reduction in intensity, which may be attributed to successful drug encapsulation within the polymeric matrix rather than chemical modification (45). The absence of additional peaks or major spectral shifts confirms that no chemical interaction, degradation, or incompatibility occurred between Exemestane and formulation excipients during nanoparticle fabrication. Minor broadening of peaks observed in the nanoparticle spectrum may result from physical interactions such as hydrogen bonding or polymer–drug entrapment within the PLGA matrix. Overall, FTIR analysis demonstrates excellent physicochemical compatibility between Exemestane, PLGA, and Poloxamer 127, indicating that the drug remained chemically stable throughout the formulation process and supporting successful encapsulation through physical incorporation rather than chemical interaction (46, 47).

#### 3.1.4 P-XRD analysis

X-ray diffraction (XRD) analysis was performed to investigate the crystalline behavior of Exemestane, formulation excipients, and the prepared Exemestane-loaded PLGA nanoparticles in order to evaluate drug–excipient compatibility and changes in physical state following nanoparticle formulation (**Figure 2b**). The diffractogram of pure Exemestane exhibited multiple sharp and intense diffraction peaks at characteristic 2θ values, confirming its highly crystalline nature. The presence of distinct peaks indicates an ordered molecular arrangement typical of crystalline drugs, which generally contributes to poor aqueous solubility (48). The XRD pattern of Poloxamer 127 (P127) showed broad and diffused peaks with low intensity, indicating its predominantly amorphous or semicrystalline structure. Similarly, PLGA displayed a broad halo pattern without sharp diffraction peaks, confirming the amorphous nature of the polymer matrix. In contrast, the XRD pattern of Exemestane-loaded PLGA nanoparticles demonstrated a significant reduction in intensity and disappearance of characteristic crystalline peaks of Exemestane. The absence of sharp drug peaks suggests successful encapsulation of Exemestane within the polymeric matrix and transformation of the drug from crystalline to an amorphous or molecularly dispersed state. The reduction in crystallinity can be attributed to uniform dispersion of drug molecules within the PLGA polymer during nanoparticle formation, preventing recrystallization of the drug (49). Importantly, no additional diffraction peaks were observed in the nanoparticle spectrum, confirming the absence of new crystalline phases or chemical interaction between drug and excipients. The conversion of Exemestane into an amorphous state within the nanoparticle system is advantageous, as amorphization enhances apparent solubility, dissolution rate, and ultimately drug release performance. These findings correlate well with FTIR results, further confirming physicochemical compatibility and successful nanoparticle formation through physical encapsulation rather than chemical modification. Overall, XRD analysis confirms that Exemestane was efficiently incorporated into the PLGA nanoparticle matrix with reduced crystallinity, demonstrating compatibility between drug and excipients and supporting improved drug delivery performance.

#### 3.1.5 TGA-DTA analysis

Thermogravimetric analysis (TGA) coupled with Differential Thermal Analysis (DTA) was performed to evaluate the thermal stability, decomposition behavior, and compatibility of Exemestane within the PLGA nanoparticle system (**Figure 2c**). The TGA thermogram demonstrated an initial negligible weight loss at lower temperatures (<100 °C), which can be attributed to the evaporation of physically adsorbed moisture and residual solvents present in the formulation. The absence of significant degradation in this temperature region confirms the thermal stability of the nanoparticle system under normal storage and processing conditions. A major weight loss was observed between approximately 300 °C and 450 °C, indicating the primary thermal degradation stage of the polymeric matrix (50). This mass loss corresponds to decomposition of PLGA polymer chains through ester bond cleavage and depolymerization processes. The gradual nature of weight reduction suggests uniform dispersion of Exemestane within the polymer matrix rather than the presence of free crystalline drug. The DTA curve exhibited characteristic endothermic and exothermic transitions corresponding to structural and decomposition events. The broad thermal transition observed at intermediate temperatures is associated with polymer relaxation and melting behavior, while the prominent peak at higher temperature regions indicates thermal degradation of the polymeric system. Importantly, no sharp independent thermal event corresponding to pure Exemestane melting was observed, suggesting successful encapsulation of the drug within the PLGA matrix and conversion into an amorphous or molecularly dispersed state.

The shift or absence of the characteristic drug thermal peak confirms the lack of chemical incompatibility between Exemestane and formulation excipients. Instead, the drug appears to be physically entrapped within the polymer network, which enhances thermal stability compared to the pure drug. Overall, TGA–DTA analysis demonstrates improved thermal stability of Exemestane after nanoparticle formulation and confirms compatibility between drug and excipients. The enhanced thermal behavior further supports successful nanoparticle formation and indicates suitability of the developed system for pharmaceutical processing and long-term storage.

#### 3.1.6 *In vitro* drug release

The *in vitro* drug release behavior of Exemestane from PLGA nanoparticles was evaluated to investigate the release kinetics and sustained delivery potential of the developed nanoparticulate system (**Figure 2d**). The release profile demonstrated a characteristic biphasic pattern consisting of an initial controlled release phase followed by a prolonged sustained release phase. An initial gradual drug release was observed during the early time intervals, which may be attributed to diffusion of drug molecules weakly associated near or at the nanoparticle surface (51, 52). Unlike conventional formulations, no significant burst release was observed, indicating efficient encapsulation of Exemestane within the PLGA polymeric matrix. The absence of burst release confirms successful nanoparticle formation and uniform drug distribution within the polymer structure. Following the initial phase, drug release proceeded in a controlled and sustained manner over the entire study period. Approximately half of the drug was released by the end of the experiment, suggesting slow polymer erosion and diffusion-controlled release behavior (52). This sustained release pattern is mainly governed by water penetration into the polymer matrix, gradual PLGA degradation, and diffusion of the entrapped drug through the polymeric network. The narrow error bars (mean ± SD, n = 3) indicate good reproducibility of the formulation and uniform nanoparticle characteristics across replicates. The steady increase in cumulative drug release without abrupt fluctuations further confirms formulation stability and controlled drug liberation (11). The sustained release behavior observed is advantageous for anticancer therapy, as prolonged drug availability can maintain therapeutic drug concentration while minimizing dosing frequency and systemic side effects. PLGA nanoparticles therefore act as an efficient reservoir system capable of regulating Exemestane release through combined diffusion and polymer degradation mechanisms (50). Overall, the developed Exemestane-loaded PLGA nanoparticles exhibited desirable controlled release characteristics, supporting their suitability as a sustained drug delivery platform for localized or long-acting therapeutic applications.

### 3.2 Optimization of *in situ* gel formation

#### 3.2.1 Impact on gelation time

The effect of formulation variables on gelation time was investigated using a Design of Experiments (DOE) approach to optimize the sol–gel transition behavior of the thermosensitive *in-situ* gel. Poloxamer A concentration (A), Poloxamer B concentration (B), and stirring time (C) were selected as independent variables. ANOVA analysis indicated that the linear model developed for gelation time was statistically significant (F-value = 3.75, p = 0.0326), confirming that formulation factors significantly influenced gel formation behavior (53). Among the variables studied, Poloxamer A was identified as the most significant factor (p = 0.0232), whereas Poloxamer B showed a non-significant effect (p = 0.3017). Stirring time exhibited moderate influence but remained statistically non-significant (p = 0.0690). The lack-of-fit was non-significant (p=0.5159), demonstrating good agreement between predicted and experimental results.

The regression equation obtained in terms of coded factors for gelation time was:


Gelation Time=2.07−0.4929A+0.2096B−0.3829C

**Figure 3:**
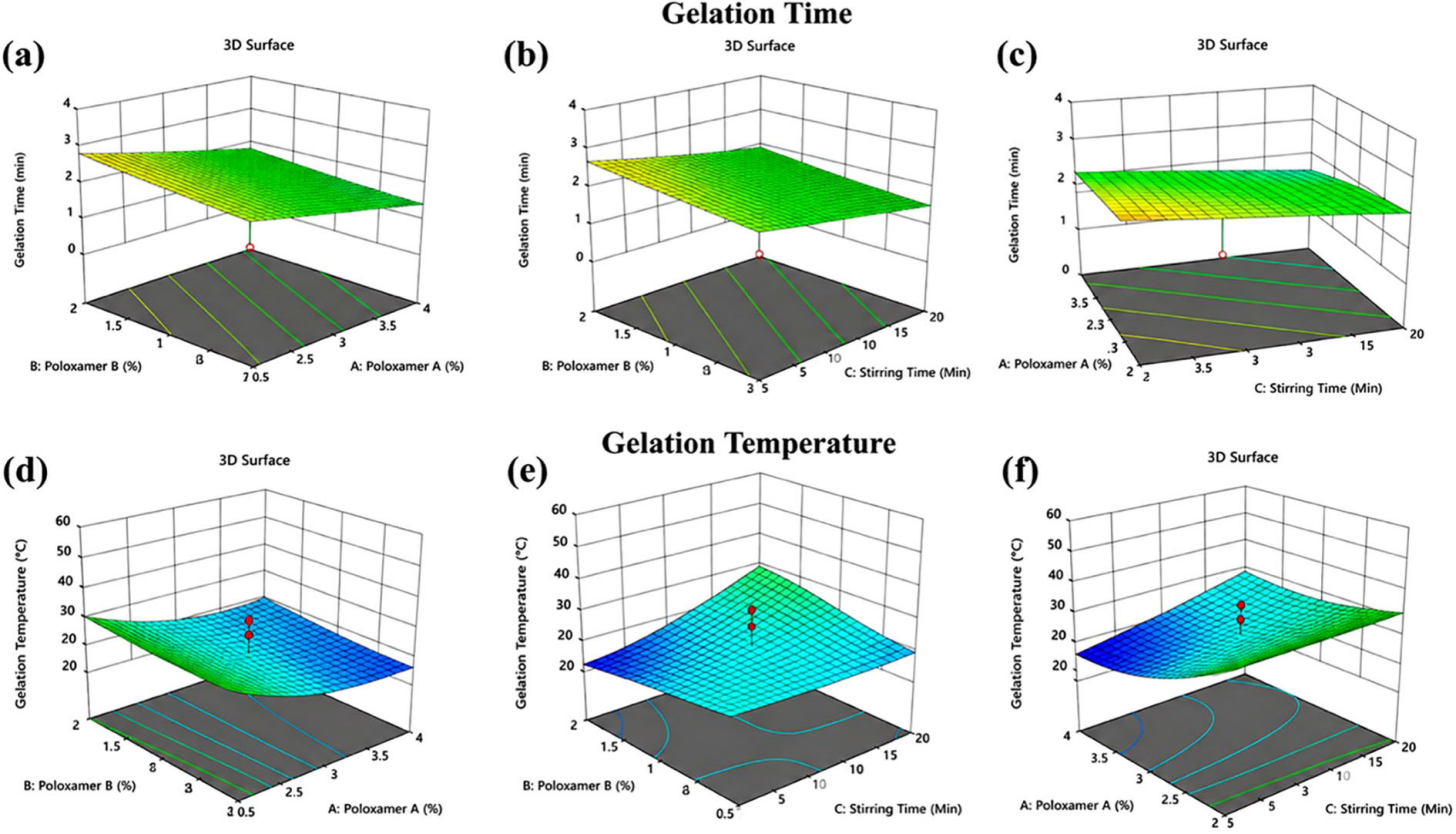
Three-dimensional response surface plots showing the effect of formulation variables on gelation time **(a–c)** and gelation temperature **(d–f)** of the thermoresponsive in situ gel, illustrating the influence and interaction of Poloxamer A concentration, Poloxamer B concentration, and stirring time during formulation optimization.

The negative coefficient of Poloxamer A indicates that increasing its concentration significantly decreases gelation time, resulting in faster sol-to-gel transition (**Figures 3a–c**). This behavior is attributed to enhanced micellar aggregation and increased hydrophobic interactions within the poloxamer matrix, which promote rapid three-dimensional network formation. Stirring time also showed a negative coefficient, suggesting that improved mixing enhances polymer hydration and uniform distribution, thereby accelerating gel formation. Conversely, Poloxamer B displayed a slight positive coefficient, indicating a marginal delay in gelation, although its effect was statistically insignificant. The optimized formulation exhibited a gelation time of 0.75 ± 0.5 min, demonstrating rapid gel formation suitable for *in-situ* drug delivery applications where quick gelation after administration is essential (54).

#### 3.2.2 Impact on gelation temperature

Gelation temperature is a critical parameter governing thermoresponsive behaviour, as the formulation must remain liquid during administration and undergo gelation near physiological temperature. Optimization using DOE revealed that the reduced quadratic model for gelation temperature was significant (F-value=2.98, p = 0.0466), confirming the suitability of the model for response prediction. Statistical analysis demonstrated that Poloxamer A concentration significantly affected gelation temperature (p = 0.0070), while Poloxamer B concentration and stirring time individually had non-significant effects. The non-significant lack-of-fit (p = 0.1868) indicated an adequate model fit.

The regression equation obtained for gelation temperature in coded factors was:


$$\begin{array}{c} \text{Gelation Temperature}=40.18-3.98A+0.058B+1.32C+0.8625AB+2.14AC+3.01BC+2.24A^{2} \end{array}$$

The strong negative coefficient for Poloxamer A confirms that increasing the polymer concentration lowers the gelation temperature. This phenomenon occurs because higher polymer content enhances micelle formation and packing density, enabling gel formation at lower temperatures. The positive coefficient of stirring time suggests a slight increase in gelation temperature due to improved polymer hydration prior to micelle aggregation. Interaction terms (AB, AC, and BC) indicate synergistic effects between formulation variables, reflecting the complex thermogelling mechanism of poloxamer-based systems. The quadratic term (A²) further suggests curvature in response behaviour at higher polymer concentrations.

The optimized formulation exhibited a gelation temperature of 38.3 ± 0.5 °C, which is close to physiological temperature, confirming appropriate thermosensitive characteristics. The close agreement between predicted and observed responses validates the robustness and predictive capability of the DOE optimization process (**Figures 3d–f**). The results clearly indicate that Poloxamer concentration (especially Poloxamer A) plays a crucial role in controlling both gelation time and gelation temperature. Increasing polymer concentration promotes rapid gel formation and lowers gelation temperature, making the system suitable for thermoresponsive *in situ* drug delivery.

#### 3.2.3 Optimization using desirability and overlay plot

**Figure 4:**
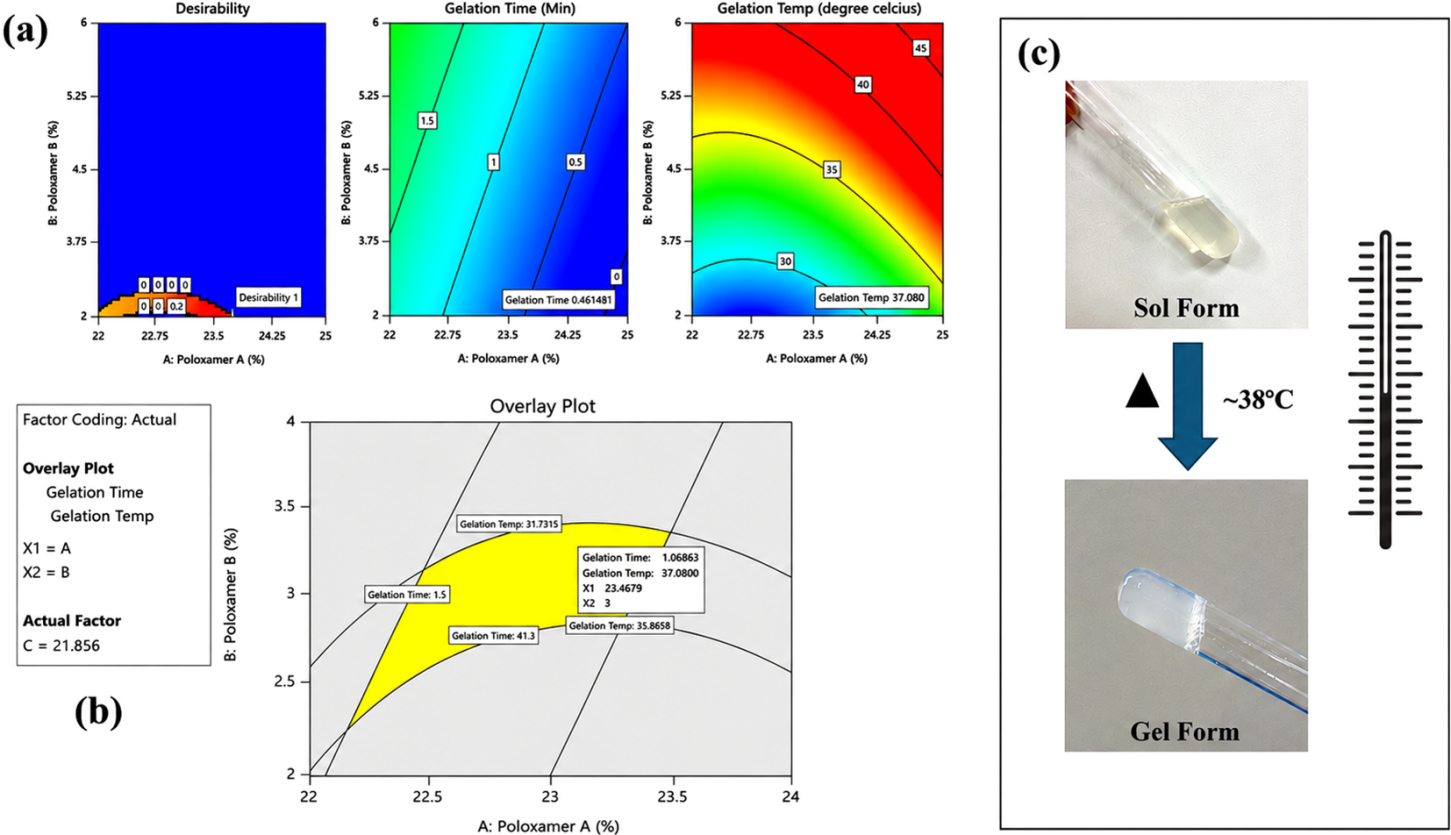
Optimization and thermoresponsive behavior of the nanoparticle-loaded in situ gel showing **(a)** desirability plots for simultaneous optimization of gelation time and gelation temperature, **(b)** overlay plot identifying the design space and optimized formulation region, and **(c)** visual sol-to-gel transition of the formulation at physiological temperature (~38 °C).

The optimization of the thermoresponsive *in situ* gel was performed using a desirability function approach (**Figure 4a**), considering gelation time and gelation temperature as critical quality attributes. The desirability plot indicated a distinct optimal region at specific concentrations of Poloxamer A and Poloxamer B, where both responses satisfied the predefined criteria. The desirability value approached unity within this region, suggesting that the selected formulation conditions are highly suitable for achieving the desired gel characteristics. The contour plots for gelation time and gelation temperature further demonstrate the influence of formulation variables. Gelation time was found to decrease with increasing Poloxamer A concentration, while gelation temperature showed a decreasing trend with increasing polymer concentration, confirming the thermosensitive behavior of the system. The overlay plot (**Figure 4b**) represents the combined effect of independent variables on both responses, highlighting a yellow region as the design space, where all constraints were simultaneously satisfied. The optimized formulation was identified at approximately Poloxamer A (23.26%) and Poloxamer B (3%), with a fixed stirring time (C ≈ 21.856 min). At this point, the predicted responses were gelation temperature and gelation time, 37-39 °C and 1–2 min, respectively. These values fall within the desired physiological range, ensuring suitability for *in situ* gel formation upon administration (**Table 3****).**

**Table 3:** Independent variables with their respective levels and selected responses (dependant variables) for formulation design and optimization of PLGA-NPs loaded in situ gel through response surface methodology- Box-Behnken model.

Levels	Independent variables		
	Poloxamer A (X1) %	Poloxamer B (X2) %	Stirring time (X3) min
High (+)	25	6	20
Medium (0)	23	4	17.5
Low (-)	21	2	15
	Dependent variables		
	Gelation time (Y1)	Gelation temperature (Y2)	
Units	Minutes	°C	
Constraints	Target range	Target range	

#### 3.2.4 Sol–gel transition behavior

The thermoresponsive behavior of the optimized formulation was visually confirmed (**Figure 4c**). The formulation existed as a free-flowing sol at room temperature, allowing ease of administration. Upon exposure to physiological temperature (~38 °C), the formulation rapidly transformed into a viscous gel, demonstrating efficient sol-to-gel transition. This phase transition is attributed to the temperature-dependent micellization of Poloxamer polymers, where increased temperature leads to dehydration of poly(propylene oxide) blocks, resulting in micelle aggregation and gel network formation. The rapid gelation at near-body temperature ensures prolonged residence time at the site of administration, which is essential for sustained drug release.

The optimized formulation was validated by comparing predicted and experimental responses under the selected conditions of Poloxamer A (23.26%), Poloxamer B (3%), and stirring time (~22 min). The predicted gelation time and temperature were 1.05 min and 37.03 °C, respectively, while the observed values were 0.75 ± 0.5 min and 38.3 ± 0.5 °C. The experimentally observed responses were in close agreement with the predicted values, confirming the validity and predictive capability of the optimization model (**Table 4**). Overall, the close agreement confirms the reliability and robustness of the optimization model, with the formulation exhibiting rapid gelation and a gelation temperature within the physiological range, making it suitable for thermoresponsive *in situ* drug delivery.

**Table 4:** Validation of the optimized thermoresponsive in-situ gel formulation showing agreement between predicted and experimentally observed responses for critical formulation variables and gelation parameters.

Factors	Predicted	Observed
Poloxamer A	23.26%	23.26%
Poloxamer B	3%	3%
Stirring time	21.856mins	22mins
Gelation time	1.05min	0.75 ± 0.5min
Gelation temperature	37.03°C	38.3 ± 0.5°C

### 3.3 Characterization of *in situ* gel

#### 3.3.1 Visual appearance, pH, gelling capacity and drug content

The optimized thermoresponsive *in situ* gel exhibited a clear, transparent, and homogeneous appearance, indicating uniform distribution of formulation components without any visible particulate matter. The pH of the optimized formulation was 4.7 ± 0.1, which lies within the physiological pH range of healthy human skin (approximately 4.5–5.5). Maintaining a slightly acidic pH helps preserve the skin’s acid mantle and barrier function. However, biological safety should be confirmed through future cytocompatibility and skin irritation studies. The formulation demonstrated immediate gelation with good gelling capacity (++), confirming its efficient sol-to-gel transition at physiological conditions, which is essential for prolonged residence at the site of administration. The drug content was 87.45 ± 5.09%, indicating satisfactory drug loading and uniform distribution within the gel matrix. The slight variation in drug content may be attributed to processing and mixing conditions; however, the values remain within acceptable limits as mentioned in **Table 5** (55). These results confirm that the developed formulation possesses suitable physicochemical properties for effective and sustained delivery of Exemestane.

**Table 5:** Physicochemical characterization of the optimized Exemestane-loaded PLGA nanoparticle incorporated thermoresponsive in-situ gel, including visual appearance, pH, gelling capacity and drug content.

Parameter	Result
Visual Appearance	Clear, transparent, homogeneous gel
pH	4.7 ± 0.1
Gelling Capacity	Immediate gelation, (++)
Drug Content (%)	87.45 ± 5.09

#### 3.3.2 Gelation temperature and time

The *in-situ* gel loaded with PLGA-NPs must have an acceptable viscosity to be delivered as a liquid via a syringe and undergo an immediate sol-gel transition. The formed gel should also retain its integrity to provide sustained local release of pharmaceuticals rather than disintegrating or eroding rapidly. *In vitro* gelation time is the time it takes for the polymer solution to transition from the liquid phase to the gel phase (56). The time of gelation of the *in-situ* nanogel was measured to be 0.75 ± 0.5 min *in vitro*. Correspondingly, the gelling temperature of the optimized batch was noticed to be 38.3 ± 0.5 °C.

#### 3.3.3 Rheological behavior of gel

**Figure 5:**
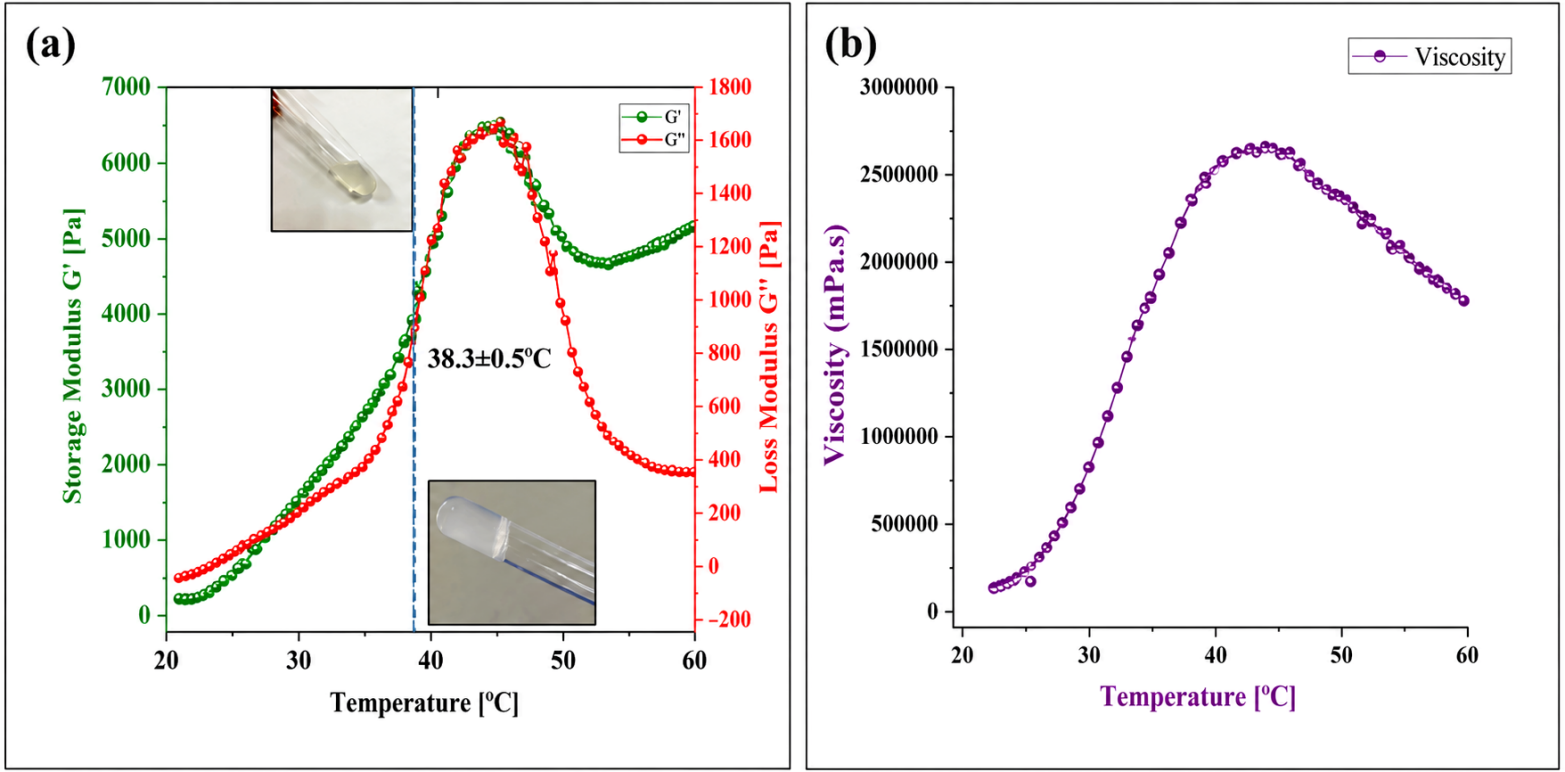
Rheological characterization of the optimized thermoresponsive in situ gel showing **(a)** temperature-dependent changes in storage modulus (G′) and loss modulus (G″) confirming sol–gel transition near physiological temperature, and **(b)** viscosity profile demonstrating thermoresponsive gelation behavior with increasing temperature.

Viscosity values were determined to establish how temperature affected the rheology of the polymer solution and gel matrix (**Figures 5a, b**). The viscosity of the polymer solution varied from 6.2 Pa·s to 36 mPa·s as the temperature rose from 25 °C to 38 °C. Analytical mechanism-based rheological experiments have shown that an aqueous solution of poloxamer 407 develops micellar due to hydrophobic interactions between the poly (propylene oxide) molecules (57). Moreover, as the temperature increases, the poly(propylene oxide) core dehydrates. In contrast, the hydrophilic poly(ethylene oxide) shell hydrates and swells, forming clear, firm gels (58).

#### 3.3.4 FTIR analysis

Fourier Transform Infrared (FTIR) spectroscopy was employed to investigate the compatibility between Exemestane, PLGA, and excipients, and to confirm the successful incorporation of the drug into the nanoparticle-loaded thermoresponsive *in situ* gel. The FTIR spectrum of pure Exemestane exhibited characteristic peaks corresponding to its functional groups as shown in **Supplementary Figure 1**. A prominent peak observed around 1735–1740 cm^−1^ is attributed to the C=O stretching of the ester/ketone group, confirming the steroidal structure of the drug. Peaks in the region of 1600–1650 cm^−1^ correspond to C=C stretching vibrations, while bands near 2950–2850 cm^−1^ indicate aliphatic C–H stretching. Additionally, weak bands in the range of 1200–1000 cm^−1^ are associated with C–O stretching vibrations. The FTIR spectrum of PLGA polymer showed a characteristic strong absorption band around 1750–1760 cm^−1^ (59), corresponding to the ester carbonyl (C=O) stretching, which is a signature peak of PLGA. Peaks observed in the range of 1080–1180 cm^−1^ are attributed to C–O–C stretching vibrations, while bands near 2990–2850 cm^−1^ correspond to –CH stretching of the polymer backbone (59, 60). In the spectrum of the physical mixture, all major characteristic peaks of Exemestane and PLGA were retained without significant shifts, indicating the absence of chemical interaction between the drug and polymer. Minor variations in peak intensity were observed, which can be attributed to simple mixing and overlapping of peaks. The FTIR spectrum of the optimized PLGA nanoparticle-loaded thermoresponsive *in situ* gel formulation demonstrated the presence of all essential functional group peaks of both drug and polymer. Notably, the carbonyl peak (~1735 cm^−1^) remained intact with slight broadening, suggesting successful encapsulation of Exemestane within the polymer matrix rather than chemical modification (55, 61). The broadening observed in the region of 3200–3500 cm^−1^ can be attributed to hydrogen bonding interactions and the presence of gel-forming polymers, indicating the formation of a structured gel network. Furthermore, no new peaks or significant peak shifts were observed in the formulation spectrum, confirming chemical stability and compatibility of Exemestane with PLGA and other excipients used in the system.

#### 3.3.5 P-XRD

X-ray diffraction (XRD) analysis was carried out to evaluate the crystalline or amorphous nature of Exemestane, excipients, PLGA nanoparticles, and the final freeze-dried *in situ* gel formulation. The diffractogram of pure Exemestane (**Supplementary Figure 2**) exhibited sharp and intense characteristic peaks, particularly in the region of 2θ = 10–25°, indicating its highly crystalline nature. These distinct diffraction peaks confirm the ordered molecular arrangement of the drug. The XRD pattern of PLGA nanoparticles (**Supplementary Figure 2b**) showed a broad halo with reduced peak intensity, suggesting the amorphous nature of PLGA. The absence of sharp peaks indicates that the polymer lacks long-range crystalline order, which is typical for PLGA. In the case of chitosan (**Supplementary Figure 2c**), a broad diffraction peak around 2θ ≈ 20° was observed, indicating its semi-crystalline nature. Similarly, Poloxamer 407 (**Supplementary Figure 2d**) and Poloxamer 188 (**Supplementary Figure 2e**) exhibited distinct sharp peaks, reflecting their crystalline characteristics. Importantly, the XRD pattern of the freeze-dried gel formulation (**Supplementary Figure 2f**) displayed a broad diffuse halo with the complete disappearance of sharp crystalline peaks of Exemestane. This indicates that the drug is no longer present in its crystalline state but is converted into an amorphous or molecularly dispersed form within the polymeric matrix. The absence of characteristic Exemestane peaks in the final formulation suggests successful encapsulation of the drug within PLGA nanoparticles and uniform dispersion within the thermoresponsive gel system. Additionally, the reduction in crystallinity may enhance the solubility and dissolution rate of Exemestane, thereby contributing to improved drug release behavior. No new diffraction peaks were observed in the formulation, confirming that no polymorphic transformation or chemical interaction occurred during formulation development.

#### 3.3.6 TGA-DTA

**Figure 6:**
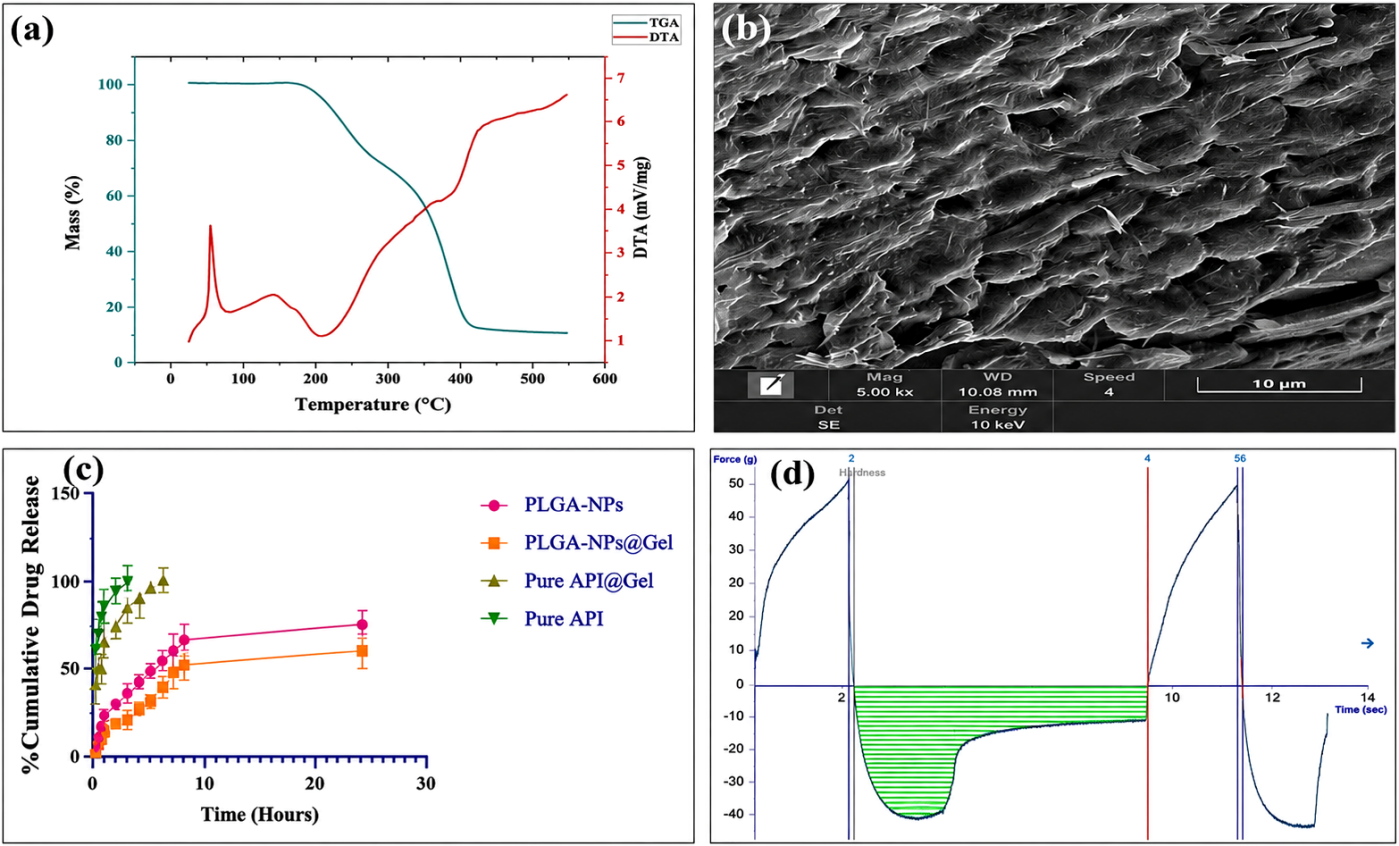
Characterization of the optimized nanoparticle-loaded thermoresponsive in situ gel showing **(a)** TGA-DTA thermograms indicating thermal stability, **(b)** FESEM micrograph illustrating surface morphology of the freeze-dried gel, **(c)** comparative in vitro drug release profile of pure Exemestane, Pure Exemestane-loaded in Gel, PLGA nanoparticles, and PLGA nanoparticle-loaded in situ gel demonstrating sustained release behavior, and **(d)** texture profile analysis showing mechanical strength and adhesiveness of the optimized gel formulation.

Thermogravimetric analysis (TGA) and differential thermal analysis (DTA) were performed to evaluate the thermal stability and decomposition behavior of the optimized formulation (**Figure 6a**). The TGA thermogram demonstrated that the formulation remained thermally stable up to approximately 200 °C, with negligible weight loss observed within this temperature range, indicating the absence of significant moisture or volatile impurities. A gradual weight loss was observed between 200 °C and 300 °C, which may be attributed to the onset of degradation of the polymeric components and partial breakdown of the formulation matrix. A major and rapid mass loss occurred between 300 °C and 400 °C, where the residual mass decreased sharply, indicating extensive thermal degradation of the PLGA polymer backbone and other organic formulation components, including the encapsulated drug. Beyond 400 °C, only a small residual mass remained, suggesting near-complete decomposition of the organic constituents. The DTA thermogram exhibited an initial endothermic event below 100 °C, attributable to the removal of physically adsorbed moisture and residual solvent. It is important to note that the glass transition temperature (Tg) of PLGA is generally reported in the range of 40–60 °C, depending on its molecular weight and lactide:glycolide ratio. Owing to the broad and subtle nature of the glass transition, and the overlap with the baseline in the DTA thermogram, a distinct Tg was not clearly resolved under the experimental conditions employed. The thermal event observed in the temperature range of 150–250 °C is therefore attributed to polymer chain relaxation and the onset of thermal degradation of the formulation components rather than the glass transition of PLGA. Furthermore, the prominent exothermic peak observed between 300 °C and 400 °C corresponds to the major thermal decomposition of the polymeric matrix. The absence of a sharp melting endotherm corresponding to crystalline Exemestane indicates that the drug is present in an amorphous or molecularly dispersed state within the PLGA matrix, which is consistent with the XRD results. Overall, the thermal analysis demonstrates that the developed formulation possesses adequate thermal stability under processing and storage conditions while undergoing thermal degradation only at elevated temperatures (30).

#### 3.3.7 FESEM analysis

The FESEM image revealed a rough, porous, and layered morphology of the freeze-dried gel, with interconnected channels formed due to the freeze-drying process as mentioned in **Figure 6b**. The surface appeared irregular and wrinkled, indicating a well-formed polymeric network. No visible crystalline drug particles were observed, suggesting uniform encapsulation of Exemestane within PLGA nanoparticles and the gel matrix. The porous structure is expected to facilitate water penetration, swelling, and controlled drug release, supporting the sustained release behavior of the formulation (62).

#### 3.3.8 *In vitro* drug release & release kinetics

The *in vitro* drug release profiles of Pure API, Pure API-loaded gel, PLGA nanoparticles, and PLGA nanoparticle-loaded thermoresponsive gel are presented in **Figure 6c**. The Pure API exhibited rapid dissolution, with nearly complete drug release occurring within 6 h due to the absence of any diffusion barrier (63). Similarly, the Pure API incorporated directly into the gel also demonstrated rapid release, reaching almost complete release within 6–8 h. This suggests that the thermoresponsive gel alone provided only limited resistance to drug diffusion for the freely dispersed Exemestane.

In contrast, Exemestane-loaded PLGA nanoparticles exhibited a sustained release profile, releasing approximately 75% of the drug over 24 h. The prolonged release is attributed to the diffusion of the drug through the PLGA polymer matrix followed by gradual polymer relaxation, which delays drug liberation.

When the PLGA nanoparticles were incorporated into the thermoresponsive gel, the release became even more controlled, with approximately 60% cumulative drug release after 24 h. The slower release can be attributed to the combined diffusion barriers provided by both the PLGA nanoparticles and the gel matrix. Initially, the drug diffuses from the nanoparticle core into the surrounding gel, after which it must further diffuse through the three-dimensional gel network before reaching the release medium. This dual-controlled release mechanism effectively reduces the diffusion rate and prolongs drug release compared with PLGA nanoparticles alone. Such sustained release is advantageous for localized breast cancer therapy, as it may maintain therapeutic drug concentrations at the target site for an extended period while minimizing burst release and reducing systemic exposure. To elucidate the release mechanism (**Supplementary Figures 3a–e**), the data were fitted to various kinetic models including zero-order, first-order, Higuchi, Hixson–Crowell, and Korsmeyer–Peppas models. The zero-order model showed a relatively poor fit (R² = 0.7550), indicating that drug release was not constant over time. The first-order model exhibited better correlation (R² = 0.9062), suggesting some dependence on drug concentration. However, a higher correlation was observed with the Higuchi model (R² = 0.9315), indicating that diffusion plays a significant role in drug release from the matrix system. The best fit was obtained with the Korsmeyer–Peppas model (R² = 0.9506), confirming its suitability for describing release from polymeric nanoparticulate systems. The release exponent (n = 0.7855) falls within the range of 0.45–0.89, indicating a non-Fickian (anomalous) transport mechanism. This suggests that drug release is governed by a combination of diffusion through the polymer matrix and polymer relaxation or erosion processes. The Hixson–Crowell model (R² = 0.8595) further supports the involvement of changes in surface area and matrix structure during drug release (64). The results confirm that incorporating exemestane into PLGA nanoparticles within a thermoresponsive *in situ* gel effectively modulates drug release, providing sustained delivery compared with immediate-release systems. This controlled release behavior is advantageous for maintaining therapeutic drug levels over an extended period, thereby enhancing efficacy and reducing dosing frequency (65).

#### 3.3.9 Texture analysis

The texture analyzer graph (**Figure 6d**) demonstrated the mechanical properties of the thermoresponsive *in situ* gel, including hardness and adhesiveness. The formulation exhibited a hardness value of approximately 23 g during the first compression cycle and 56 g during the second cycle, indicating good gel strength and structural integrity. The increase in hardness in the second cycle suggests enhanced gel firmness after initial deformation, confirming the stability of the polymeric network. The negative area observed between the two compression cycles represents adhesiveness, indicating the work required to detach the probe from the gel surface. The formulation showed a significant negative peak, suggesting good mucoadhesive properties, which are desirable for prolonged retention at the site of application. Overall, the texture profile indicates that the gel possesses adequate mechanical strength, elasticity, and adhesiveness, supporting its suitability for *in situ* gelation and sustained drug delivery applications.

#### 3.3.10 Gel erosion study

**Figure 7:**
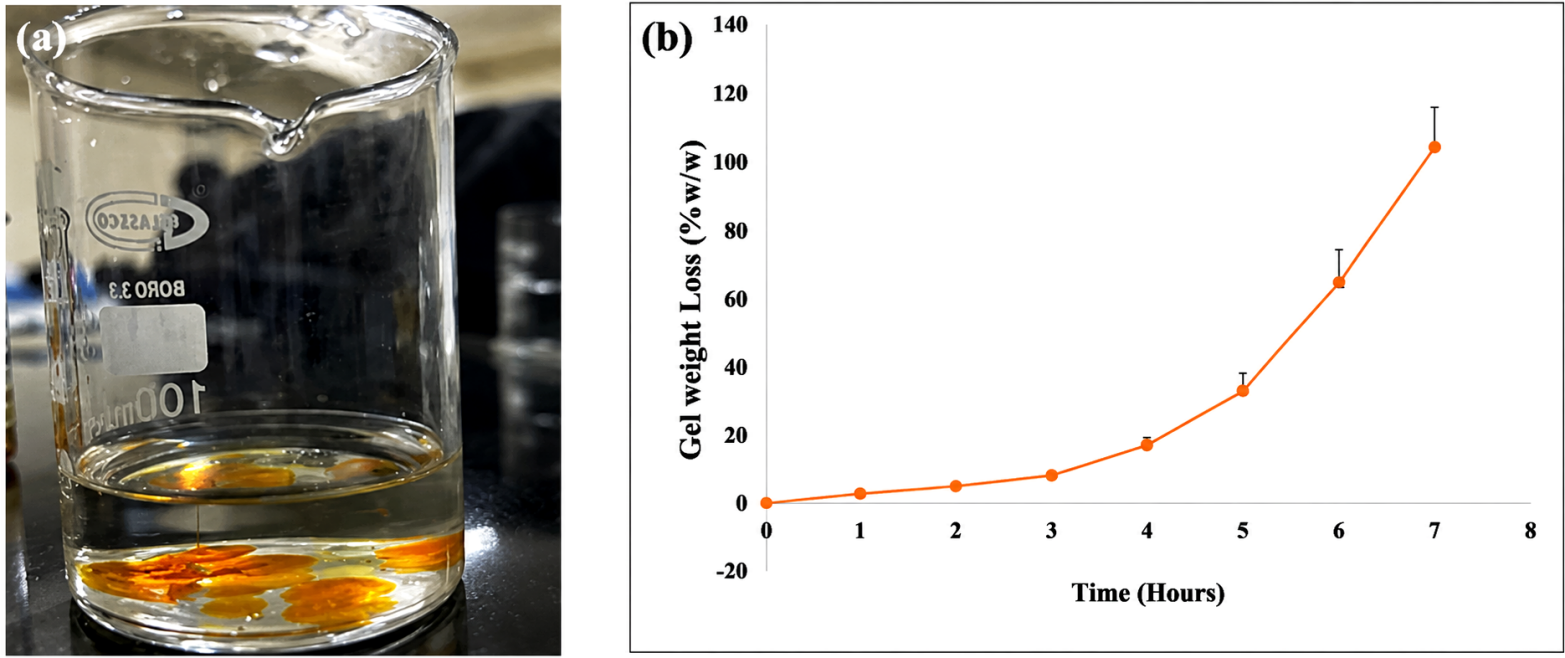
Gel erosion behavior of the optimized thermoresponsive in situ gel showing **(a)** visual representation of gel integrity during erosion study and **(b)** time-dependent percentage gel weight loss under physiological conditions, demonstrating controlled erosion of the gel matrix.

The gel erosion behavior of the thermoresponsive *in situ* gel was evaluated over a period of 7 hours, and the results demonstrated a clear time-dependent increase in percentage weight loss (67). At the initial time point (0 h), no erosion was observed, indicating the formation of a stable and intact gel matrix upon exposure to physiological conditions (**Figures 7a, b**). During the early phase, the formulation exhibited minimal and controlled erosion, with weight loss values of 2.87 ± 0.20% at 1 h, 4.89 ± 0.40% at 2 h, and 8.14 ± 0.90% at 3 h, suggesting the presence of a tightly packed polymeric network that resists immediate disintegration. A significant increase in erosion was observed after 4 h, with 16.89 ± 1.89% weight loss, followed by a sharp rise to 32.94 ± 4.87% at 5 h and 64.76 ± 9.30% at 6 h, indicating progressive weakening and hydration of the gel matrix. Complete erosion was achieved at 7 h (104.09 ± 11.27%), which may be attributed to extensive swelling, polymer chain relaxation, and eventual disintegration of the gel structure. The erosion profile thus follows a biphasic pattern, characterized by an initial slow surface erosion phase (0–3 h) followed by rapid bulk erosion (4–7 h). The relatively low standard deviation values during the initial phase indicate uniform gel formation, whereas increased variability at later stages reflects matrix breakdown. The incorporation of PLGA nanoparticles likely contributed to the delayed erosion by reinforcing the gel network and providing resistance to early degradation (53). Overall, the observed controlled erosion behavior supports a sustained drug release mechanism, where drug diffusion predominates initially, followed by erosion-assisted release at later stages.

#### 3.3.11 HET CAM assay

**Figure 8:**
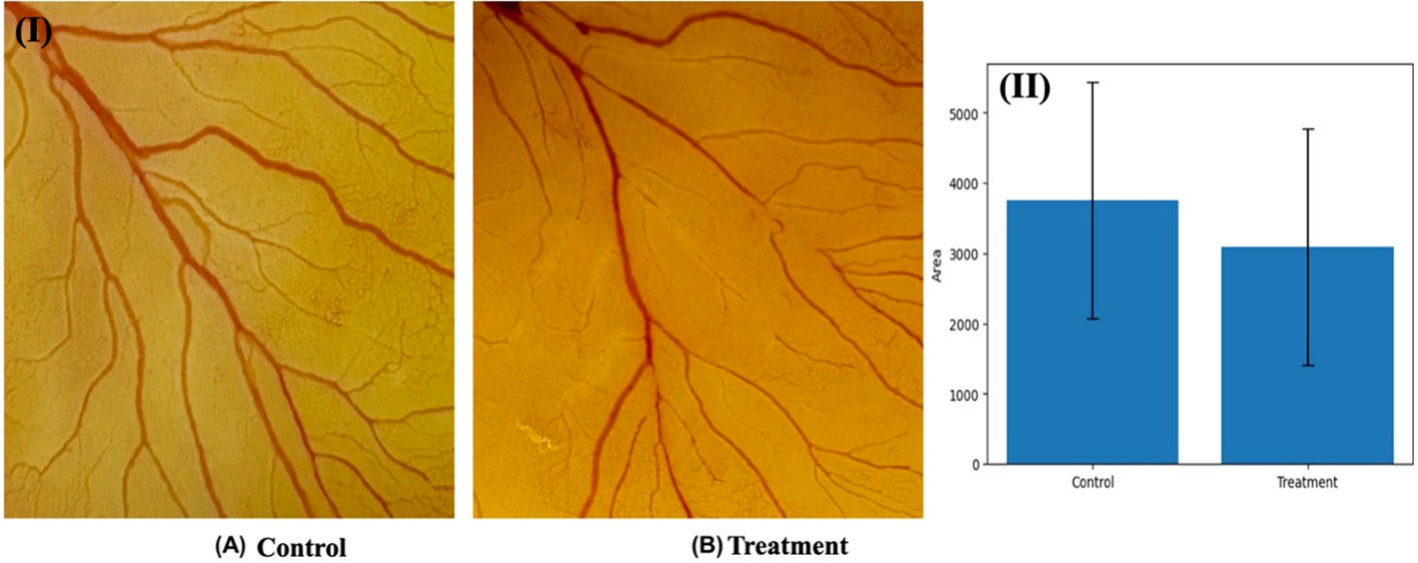
HET-CAM assay demonstrating the anti-angiogenic potential of the optimized Exemestane-loaded PLGA nanoparticle in situ gel showing (I) representative chorioallantoic membrane images of **(A)** control and **(B)** treatment groups, and (II) quantitative analysis of vascular area indicating reduced angiogenesis in the treated group.

The anti-angiogenic potential of the developed thermoresponsive *in situ* gel loaded with PLGA nanoparticles of exemestane was evaluated using the HET-CAM assay. The Exemestane concentration of 2 mg/mL was selected based on previously reported injectable thermoresponsive formulations of hydrophobic anticancer agents, where similar concentrations provided an appropriate balance between drug loading, formulation stability, and sustained release characteristics while maintaining sink conditions during *in vitro* release studies (34). Quantitative analysis of vascular area revealed a noticeable reduction in angiogenesis in the treatment group compared to the control as shown in **Figure 8a**. The total vascular area in the control group, calculated from multiple images, was found to be ~269,901, 264,398, and 236,410 units, indicating a dense and well-developed vascular network (**Figure 8b**). In contrast, the treatment group exhibited reduced vascular areas of 234,781, 290,005, and 233,337 units, suggesting a comparatively lower degree of vascularization (35). Although some variability was observed between samples, the overall trend indicates a reduction in vessel density and branching in the treated CAM, which is consistent with the visual observations of diminished microvascular networks. This anti-angiogenic effect can be attributed to the sustained release of exemestane from PLGA nanoparticles, enabling prolonged drug exposure at the site of action. Additionally, the thermoresponsive *in situ* gel enhances localized retention and controlled delivery, thereby improving therapeutic efficiency. The moderate decrease in vascularization suggests that the formulation effectively inhibits angiogenesis without causing severe vascular damage, which is crucial for ensuring biocompatibility. Mechanistically, exemestane may suppress estrogen-mediated signaling pathways involved in endothelial cell proliferation and neovascularization. The nanoparticulate delivery system further facilitates enhanced interaction with the CAM tissue, contributing to the observed biological response (33, 46). These findings demonstrate that the developed formulation possesses promising anti-angiogenic activity, aligning with its sustained release profile and supporting its potential application for localized breast cancer therapy with reduced systemic exposure.

#### 3.3.12 Stability studies

Stability studies (**Table 6**) indicated that the developed thermoresponsive *in situ* gel remained physically stable over a period of 3 months, with no observable changes in appearance or clarity. The gelation time remained consistent, with an average value of 0.745 ± 0.017 min, showing minimal variation (%CV< 3%). Similarly, the gelation temperature was maintained within a narrow range (38.4 ± 0.31 °C), indicating stable Thermoresponsive behavior. Statistical analysis using one-way ANOVA revealed no significant differences (p > 0.05) in gelation parameters across different time points. These results confirm the robustness and stability of the formulation under storage conditions.

**Table 6:** Accelerated stability evaluation of the optimized thermoresponsive in-situ gel formulation over three months showing changes in appearance, gelation time and gelation temperature.

Time points (months)	Appearance/clarity	Gelation time (min)	Gelation temperature (°C)
0	+ + +	0.75 ± 0.5	38.3 ± 0.5
1	+ + +	0.72 ± 0.4	38.2± 0.2
2	+ + +	0.76 ± 0.8	38.9± 0.5
3	+ + +	0.75 ± 0.2	38.2± 0.3

## 4 Conclusion

In conclusion, the present work successfully demonstrates the development of a rationally designed hybrid drug delivery system comprising Exemestane-loaded PLGA nanoparticles incorporated within a thermoresponsive *in-situ* gel for sustained and localized delivery. The developed formulation combined the advantages of polymeric nanoparticles and thermosensitive gel systems to achieve a dual-controlled release platform capable of minimizing burst release while prolonging drug retention and release at the target site. The optimized system exhibited favorable nanoscale characteristics, high encapsulation efficiency, suitable gelation behavior at physiological temperature, desirable rheological properties, controlled erosion, and sustained drug release over 24 hours, highlighting the synergistic role of nanoparticle diffusion and gel matrix relaxation in modulating release behavior. Physicochemical, structural, and thermal characterization confirmed successful drug incorporation, compatibility among formulation components, and enhanced stability of the developed system. Importantly, the observed anti-angiogenic response in the HET-CAM model further supports the formulation’s therapeutic promise, while stability studies confirmed its robustness under storage conditions. Taken together, these findings demonstrate that embedding Exemestane-loaded PLGA nanoparticles within a thermoresponsive *in situ* gel provides a promising localized drug delivery platform with favourable physicochemical characteristics and sustained drug release. The developed hybrid system may enhance local drug retention and prolong drug release, thereby supporting its potential for localized delivery of hydrophobic anticancer agents. However, further *in vitro* biological evaluation and *in vivo* studies are required to establish its therapeutic efficacy and safety.

## 5 Limitation of the study

The present study was limited to formulation development and physicochemical characterization. Although the developed injectable thermoresponsive PLGA nanoparticle-loaded *in situ* gel demonstrated favorable gelation characteristics, sustained drug release, and preliminary anti-angiogenic potential, biological evaluation using breast cancer and normal mammary cell lines, as well as *in vivo* therapeutic studies, were beyond the scope of this work. Future investigations will focus on cytocompatibility, anticancer efficacy, pharmacokinetics, and *in vivo* validation to further establish the safety and therapeutic potential of the developed formulation.

## Data Availability

The original contributions presented in the study are included in the article/**Supplementary Material**. Further inquiries can be directed to the corresponding author.
