# Supplementary material for: Development and characterization of an injectable thermoresponsive PLGA nanoparticle-loaded in situ gel for sustained exemestane delivery
Source: Front Oncol. 2026 Jul 23;16:1905253. doi: 10.3389/fonc.2026.1905253 (PMC13441772; doi:10.3389/fonc.2026.1905253)
Supplement: Supplementary file 1 [file DataSheet1.docx]

**Thermoresponsive In Situ Gel Incorporating PLGA Nanoparticles for Sustained Exemestane Delivery in Breast Cancer Therapy**

**Supplementary Data**

**----Exemestane**

**----PLGA-NPs**

**----P407**

**----P188**

**----Freeze-dried Gel**

***Figure S1:*** *FTIR spectra of pure Exemestane, Exemestane-loaded PLGA nanoparticles (PLGA-NPs), Poloxamer 407 (P407), Poloxamer 188 (P188), and the freeze-dried optimized in situ gel formulation showing characteristic functional group peaks and confirming drug–excipient compatibility and successful incorporation of Exemestane within the hybrid nanoparticle-loaded thermoresponsive gel system*

***Figure S2:*** *Powder X-ray diffraction (P-XRD) patterns of (a) pure Exemestane, (b) Exemestane-loaded PLGA nanoparticles, (c) chitosan, (d) Poloxamer 407, (e) Poloxamer 188, and (f) freeze-dried optimized in situ gel formulation showing reduced crystallinity and amorphous dispersion of Exemestane within the nanoparticle-loaded thermoresponsive gel system*

***Figure S3:*** *Drug release kinetic modeling of Exemestane release from the optimized PLGA nanoparticle-loaded thermoresponsive in situ gel fitted to (a) Zero-order, (b) First-order, (c) Higuchi, (d) Korsmeyer–Peppas, and (e) Hixson–Crowell models, indicating a non-Fickian diffusion-controlled release mechanism with the best fit observed for the Korsmeyer–Peppas model*
